# (*E*)-1-Ferrocenyl-3-[2-(2-hy­droxy­eth­oxy)phen­yl]prop-2-en-1-one

**DOI:** 10.1107/S1600536813003395

**Published:** 2013-02-09

**Authors:** S. Paramasivam, Santhanagopalan Purushothaman, P. R. Seshadri, Raghavachary Raghunathan

**Affiliations:** a Post Graduate and Research Department of Physics, Agurchand Manmull Jain College, Chennai 600 114, India; b Department of Organic Chemistry, University of Madras, Guindy Campus, Chennai 600 025, India

## Abstract

In the title compound, [Fe(C_5_H_5_)(C_16_H_15_O_3_)], the cyclo­penta­dienyl rings are in an eclipsed conformation and the benzene ring makes dihedral angles of 10.84 (9) and 12.35 (9)°, respectively, with the substituted and unsubstituted cyclo­penta­dienyl rings. In the crystal, mol­ecules form inversion dimers through pairs of O—H⋯O hydrogen bonds. Weak C—H⋯O hydrogen bonds are observed between the dimers.

## Related literature

For the biological activity of ferrocenyl derivatives, see: Jaouen *et al.* (2004 ▶); Fouda *et al.* (2007 ▶); Biot *et al.* (2004 ▶); Edwards *et al.* (1975 ▶). For a related structure, see: Zora *et al.* (2006 ▶).
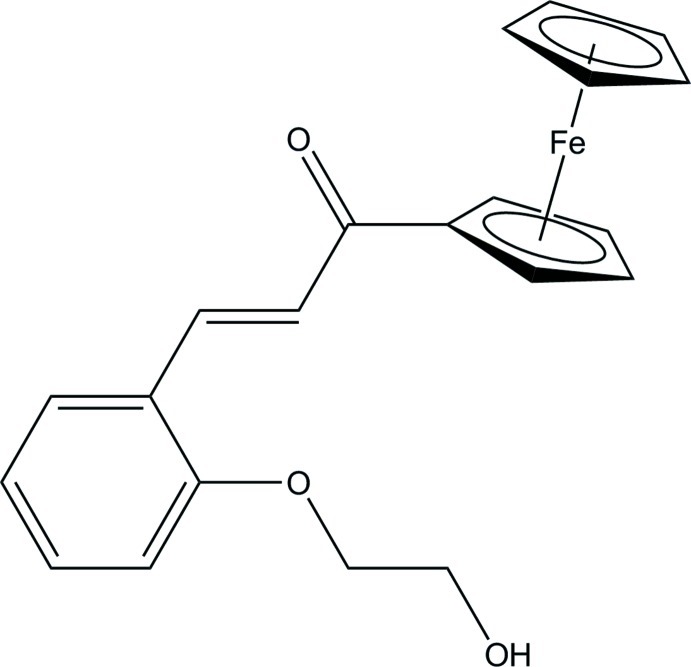


## Experimental

### 

#### Crystal data

[Fe(C_5_H_5_)(C_16_H_15_O_3_)]
*M* *_r_* = 376.22
Monoclinic, 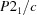

*a* = 12.5649 (10) Å
*b* = 19.0531 (14) Å
*c* = 7.4930 (6) Å
β = 103.932 (3)°
*V* = 1741.1 (2) Å^3^
*Z* = 4
Mo *K*α radiation
μ = 0.88 mm^−1^
*T* = 298 K
0.20 × 0.20 × 0.20 mm

#### Data collection

Bruker SMART APEXII area-detector diffractometer
16289 measured reflections
4310 independent reflections
3510 reflections with *I* > 2σ(*I*)
*R*_int_ = 0.045

#### Refinement

*R*[*F* ^2^ > 2σ(*F* ^2^)] = 0.035
*wR*(*F* ^2^) = 0.096
*S* = 1.06
4310 reflections
228 parameters
H-atom parameters constrained
Δρ_max_ = 0.29 e Å^−3^
Δρ_min_ = −0.28 e Å^−3^

### 

Data collection: *APEX2* (Bruker, 2008 ▶); cell refinement: *SAINT* (Bruker, 2008 ▶); data reduction: *SAINT*; program(s) used to solve structure: *SHELXS97* (Sheldrick, 2008 ▶); program(s) used to refine structure: *SHELXL97* (Sheldrick, 2008 ▶); molecular graphics: *ORTEP-3 for Windows* (Farrugia, 2012 ▶) and *PLATON* (Spek, 2009 ▶); software used to prepare material for publication: *SHELXL97*, *PLATON* (Spek, 2009 ▶) and *publCIF* (Westrip, 2010 ▶).

## Supplementary Material

Crystal structure: contains datablock(s) I, global. DOI: 10.1107/S1600536813003395/is5242sup1.cif

Structure factors: contains datablock(s) I. DOI: 10.1107/S1600536813003395/is5242Isup2.hkl

Additional supplementary materials:  crystallographic information; 3D view; checkCIF report

## Figures and Tables

**Table 1:** Hydrogen-bond geometry (Å, °)

*D*—H⋯*A*	*D*—H	H⋯*A*	*D*⋯*A*	*D*—H⋯*A*
O1—H1⋯O3^i^	0.88	1.98	2.841 (2)	165
C7—H7⋯O3^ii^	0.93	2.56	3.409 (3)	152

Symmetry codes: (i) 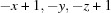; (ii) 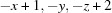.

**Fig. 1.**
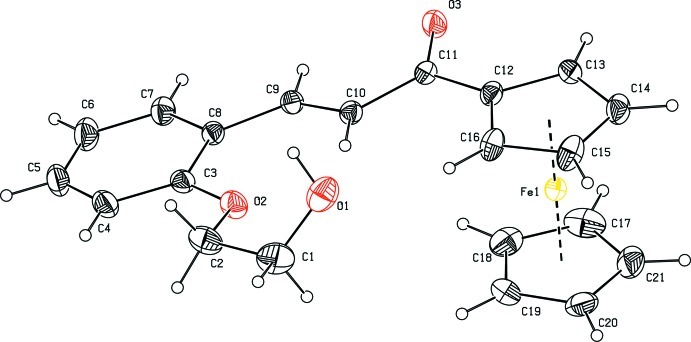
The molecular structure of the title compound, showing the atom-numbering scheme and displacement ellipsoids drawn at the 20% probability level.

## supplementary crystallographic information

### Comment

Ferrocenyl derivatives exhibit antitumor (Jaouen *et al.*, 2004), antibacterial (Fouda *et al.*, 2007), antifungal and antimalarial (Biot *et al.*, 2004) activities. It was proved that the replacement of the aromatic group by the ferrocenyl moiety in penicillins and cephalosporins could improve their antibiotic activity (Edwards *et al.*, 1975). Against this background, the title compound was chosen for X-ray structure analysis (Fig. 1).

In the title compound, the benzene ring makes dihedral angles of 10.84 (9) and 12.35 (9)°, respectively, with the substituted and unsubstituted cyclopentadienyl (Cp) rings of the ferrocenyl unit. In ferrocenyl unit, the two Cp rings are planar and are parallel to each other with a dihedral angle of 1.56 (9)° between them. The Fe atom lies in the middle of the two planes of Cp rings. The distances of the Fe1 atom from the centroids of the substituted and unsubstituted cyclopentadienyl rings are 1.646 (10) and 1.650 (12) Å, respectively. The *Cg*1—Fe1—*Cg*2 angle is 179.21 (5)°, where *Cg*1 and *Cg*2 are the centroids of substituted and unsubstituted Cp rings, respectively. The C—C bond distances in the Cp rings range from 1.381 (4) to 1.429 (3) Å, while Fe—C bond lengths range between 2.023 (1) and 2.058 (2) Å and all of which are as expected (Zora *et al.*, 2006). The torsion angles O1—C1—C2—O2 and O3—C11—C12—C13 [56.6 (3)° and -12.5 (3)°, respectively] indicate the bent conformation of the molecule. The crystal packing features O—H···O and weak C—H···O hydrogen bonds (Table 1).

### Experimental

A solution of acetylferrocene (3 g, 1.0 mmol) in ethanol (20 ml) was cooled to 0 °C and a solution of sodium hydroxide (0.48 g, 12.0 mmol) in water (2 ml) was added drop wise under vigorous stirring for 10 minutes. To the above mixture 2-(2-hydroxyethoxy)benzaldehyde (2.0 g, 12.0 mmol) in ethanol (10 ml) was added and stirred for 3 h in room temperature. Then, the reaction mixture was quenched in crushed ice and the solid obtained was filtered using Buchner funnel. The crude product was then subjected to column chromatography using hexane/ethyl acetate mixture (7:3) as eluent.

### Refinement

Hydrogen atoms were positioned geometrically and allowed to ride on their parent atoms, with C—H = 0.93–0.97 Å and O—H = 0.88 Å, and with *U*_iso_(H) = 1.5*U*_eq_(C) for methyl H atoms, 1.2*U*_eq_(C) for other C-bound H atoms and 1.5*U*_eq_(O) for the hydroxyl H atom.

### Crystal data

**Table:**

[Fe(C_5_H_5_)(C_16_H_15_O_3_)]	*F*(000) = 784
*M**_r_* = 376.22	Monoclinic
Monoclinic, *P*2_1_/*c*	*D*_x_ = 1.435 Mg m^−^^3^
Hall symbol: -P 2ybc	Mo *K*α radiation, λ = 0.71073 Å
*a* = 12.5649 (10) Å	Cell parameters from 4310 reflections
*b* = 19.0531 (14) Å	θ = 1.7–28.3°
*c* = 7.4930 (6) Å	µ = 0.88 mm^−^^1^
β = 103.932 (3)°	*T* = 298 K
*V* = 1741.1 (2) Å^3^	Block, colourless
*Z* = 4	0.20 × 0.20 × 0.20 mm

### Data collection

**Table:**

Bruker SMART APEXII area-detector diffractometer	3510 reflections with *I* > 2σ(*I*)
Radiation source: fine-focus sealed tube	*R*_int_ = 0.045
Graphite monochromator	θ_max_ = 28.3°, θ_min_ = 1.7°
ω and φ scans	*h* = −13→16
16289 measured reflections	*k* = −24→25
4310 independent reflections	*l* = −9→9

### Refinement

**Table:**

Refinement on *F*^2^	Primary atom site location: structure-invariant direct methods
Least-squares matrix: full	Secondary atom site location: difference Fourier map
*R*[*F*^2^ > 2σ(*F*^2^)] = 0.035	Hydrogen site location: inferred from neighbouring sites
*wR*(*F*^2^) = 0.096	H-atom parameters constrained
*S* = 1.06	*w* = 1/[σ^2^(*F*_o_^2^) + (0.044*P*)^2^ + 0.321*P*] where *P* = (*F*_o_^2^ + 2*F*_c_^2^)/3
4310 reflections	(Δ/σ)_max_ < 0.001
228 parameters	Δρ_max_ = 0.29 e Å^−^^3^
0 restraints	Δρ_min_ = −0.28 e Å^−^^3^

### Special details

**Table:**

Geometry. All e.s.d.'s (except the e.s.d. in the dihedral angle between two l.s. planes) are estimated using the full covariance matrix. The cell e.s.d.'s are taken into account individually in the estimation of e.s.d.'s in distances, angles and torsion angles; correlations between e.s.d.'s in cell parameters are only used when they are defined by crystal symmetry. An approximate (isotropic) treatment of cell e.s.d.'s is used for estimating e.s.d.'s involving l.s. planes.
Refinement. Refinement of *F*^2^ against ALL reflections. The weighted *R*-factor *wR* and goodness of fit *S* are based on *F*^2^, conventional *R*-factors *R* are based on *F*, with *F* set to zero for negative *F*^2^. The threshold expression of *F*^2^ > σ(*F*^2^) is used only for calculating *R*-factors(gt) *etc*. and is not relevant to the choice of reflections for refinement. *R*-factors based on *F*^2^ are statistically about twice as large as those based on *F*, and *R*- factors based on ALL data will be even larger.

### Fractional atomic coordinates and isotropic or equivalent isotropic displacement parameters (Å^2^)

**Table:**

	*x*	*y*	*z*	*U*_iso_*/*U*_eq_	
C1	0.5017 (2)	0.19106 (15)	0.1792 (4)	0.0844 (8)	
H1A	0.4927	0.2107	0.0571	0.101*	
H1B	0.5491	0.2224	0.2651	0.101*	
C2	0.3913 (2)	0.18912 (13)	0.2245 (3)	0.0687 (6)	
H2A	0.3620	0.2362	0.2247	0.082*	
H2B	0.3402	0.1612	0.1345	0.082*	
C3	0.32089 (15)	0.14956 (9)	0.4781 (2)	0.0448 (4)	
C4	0.21972 (18)	0.18139 (11)	0.4123 (3)	0.0601 (5)	
H4	0.2076	0.2092	0.3075	0.072*	
C5	0.13711 (18)	0.17179 (13)	0.5021 (4)	0.0689 (6)	
H5	0.0694	0.1931	0.4568	0.083*	
C6	0.15347 (17)	0.13110 (14)	0.6575 (3)	0.0665 (6)	
H6	0.0977	0.1254	0.7185	0.080*	
C7	0.25349 (16)	0.09891 (10)	0.7221 (3)	0.0526 (5)	
H7	0.2642	0.0712	0.8271	0.063*	
C8	0.33932 (14)	0.10650 (9)	0.6351 (2)	0.0399 (4)	
C9	0.44013 (13)	0.06738 (9)	0.7103 (2)	0.0396 (4)	
H9	0.4388	0.0402	0.8129	0.048*	
C10	0.53325 (13)	0.06421 (9)	0.6567 (2)	0.0404 (4)	
H10	0.5416	0.0911	0.5572	0.048*	
C11	0.62388 (14)	0.01847 (9)	0.7532 (2)	0.0402 (4)	
C12	0.72924 (13)	0.02332 (9)	0.7018 (2)	0.0420 (4)	
C13	0.82896 (14)	−0.00662 (10)	0.8068 (3)	0.0494 (4)	
H13	0.8358	−0.0356	0.9087	0.059*	
C14	0.91522 (16)	0.01531 (12)	0.7295 (3)	0.0582 (5)	
H14	0.9889	0.0038	0.7722	0.070*	
C15	0.86994 (16)	0.05775 (14)	0.5762 (3)	0.0618 (6)	
H15	0.9090	0.0785	0.4993	0.074*	
C16	0.75584 (15)	0.06370 (12)	0.5579 (2)	0.0524 (5)	
H16	0.7069	0.0892	0.4686	0.063*	
C17	0.8640 (3)	0.13448 (14)	1.0708 (3)	0.0789 (8)	
H17	0.8730	0.1060	1.1741	0.095*	
C18	0.7635 (2)	0.16178 (13)	0.9658 (4)	0.0694 (7)	
H18	0.6943	0.1543	0.9866	0.083*	
C19	0.7877 (2)	0.20164 (12)	0.8261 (4)	0.0678 (6)	
H19	0.7370	0.2260	0.7366	0.081*	
C20	0.8997 (2)	0.19941 (12)	0.8411 (4)	0.0699 (6)	
H20	0.9365	0.2218	0.7635	0.084*	
C21	0.9477 (2)	0.15830 (14)	0.9908 (4)	0.0736 (7)	
H21	1.0220	0.1482	1.0313	0.088*	
O1	0.55410 (15)	0.12565 (11)	0.1848 (3)	0.0782 (5)	
H1	0.5047	0.0927	0.1474	0.117*	
O2	0.40793 (11)	0.15843 (7)	0.40196 (18)	0.0548 (3)	
O3	0.61383 (11)	−0.02233 (8)	0.8744 (2)	0.0571 (4)	
Fe1	0.836512 (18)	0.100494 (12)	0.80775 (3)	0.03666 (9)	

### Atomic displacement parameters (Å^2^)

**Table:**

	*U*^11^	*U*^22^	*U*^33^	*U*^12^	*U*^13^	*U*^23^
C1	0.103 (2)	0.0783 (18)	0.0778 (16)	−0.0097 (15)	0.0332 (15)	0.0336 (14)
C2	0.0914 (17)	0.0574 (13)	0.0560 (12)	0.0086 (12)	0.0153 (11)	0.0243 (10)
C3	0.0480 (10)	0.0372 (9)	0.0448 (9)	0.0023 (7)	0.0027 (7)	−0.0046 (7)
C4	0.0599 (13)	0.0495 (11)	0.0606 (12)	0.0114 (9)	−0.0060 (10)	0.0000 (9)
C5	0.0438 (11)	0.0668 (14)	0.0871 (17)	0.0170 (10)	−0.0018 (11)	−0.0118 (13)
C6	0.0416 (11)	0.0781 (16)	0.0801 (15)	0.0070 (10)	0.0148 (10)	−0.0087 (13)
C7	0.0430 (10)	0.0591 (12)	0.0563 (11)	0.0033 (8)	0.0129 (9)	−0.0027 (9)
C8	0.0376 (9)	0.0397 (9)	0.0401 (9)	0.0008 (7)	0.0049 (7)	−0.0048 (7)
C9	0.0403 (9)	0.0401 (9)	0.0369 (8)	0.0001 (7)	0.0062 (7)	0.0017 (7)
C10	0.0382 (9)	0.0427 (9)	0.0391 (8)	−0.0022 (7)	0.0069 (7)	0.0030 (7)
C11	0.0364 (8)	0.0406 (9)	0.0414 (8)	−0.0028 (7)	0.0050 (7)	−0.0017 (7)
C12	0.0357 (8)	0.0444 (9)	0.0446 (9)	−0.0029 (7)	0.0070 (7)	−0.0090 (7)
C13	0.0420 (10)	0.0387 (9)	0.0651 (12)	0.0055 (7)	0.0081 (9)	−0.0057 (8)
C14	0.0390 (10)	0.0648 (13)	0.0709 (13)	0.0059 (9)	0.0136 (9)	−0.0197 (11)
C15	0.0453 (11)	0.0962 (17)	0.0485 (11)	−0.0104 (11)	0.0202 (9)	−0.0186 (11)
C16	0.0413 (10)	0.0805 (14)	0.0345 (9)	−0.0070 (9)	0.0071 (7)	−0.0078 (9)
C17	0.136 (3)	0.0599 (14)	0.0382 (11)	−0.0018 (15)	0.0168 (13)	−0.0121 (10)
C18	0.0701 (15)	0.0620 (14)	0.0872 (16)	−0.0094 (11)	0.0408 (13)	−0.0304 (12)
C19	0.0680 (15)	0.0461 (12)	0.0873 (16)	0.0113 (10)	0.0148 (12)	0.0026 (11)
C20	0.0710 (15)	0.0470 (12)	0.0943 (17)	−0.0173 (11)	0.0252 (13)	−0.0041 (12)
C21	0.0580 (13)	0.0725 (16)	0.0782 (16)	−0.0002 (12)	−0.0072 (12)	−0.0310 (13)
O1	0.0659 (10)	0.0967 (13)	0.0755 (11)	−0.0164 (10)	0.0240 (9)	0.0026 (10)
O2	0.0587 (8)	0.0565 (8)	0.0475 (7)	0.0058 (6)	0.0097 (6)	0.0163 (6)
O3	0.0449 (7)	0.0590 (8)	0.0663 (8)	0.0020 (6)	0.0111 (6)	0.0228 (7)
Fe1	0.03431 (14)	0.03993 (15)	0.03509 (14)	−0.00033 (9)	0.00705 (10)	−0.00048 (9)

### Geometric parameters (Å, º)

**Table:**

C1—O1	1.406 (3)	C13—C14	1.409 (3)
C1—C2	1.505 (4)	C13—Fe1	2.0429 (19)
C1—H1A	0.9700	C13—H13	0.9300
C1—H1B	0.9700	C14—C15	1.408 (3)
C2—O2	1.421 (2)	C14—Fe1	2.0579 (19)
C2—H2A	0.9700	C14—H14	0.9300
C2—H2B	0.9700	C15—C16	1.412 (3)
C3—O2	1.361 (2)	C15—Fe1	2.0496 (19)
C3—C4	1.387 (3)	C15—H15	0.9300
C3—C8	1.406 (3)	C16—Fe1	2.0271 (18)
C4—C5	1.378 (3)	C16—H16	0.9300
C4—H4	0.9300	C17—C21	1.405 (4)
C5—C6	1.373 (4)	C17—C18	1.417 (4)
C5—H5	0.9300	C17—Fe1	2.024 (2)
C6—C7	1.377 (3)	C17—H17	0.9300
C6—H6	0.9300	C18—C19	1.385 (3)
C7—C8	1.395 (3)	C18—Fe1	2.033 (2)
C7—H7	0.9300	C18—H18	0.9300
C8—C9	1.461 (2)	C19—C20	1.385 (4)
C9—C10	1.327 (2)	C19—Fe1	2.037 (2)
C9—H9	0.9300	C19—H19	0.9300
C10—C11	1.477 (2)	C20—C21	1.381 (4)
C10—H10	0.9300	C20—Fe1	2.037 (2)
C11—O3	1.225 (2)	C20—H20	0.9300
C11—C12	1.468 (2)	C21—Fe1	2.030 (2)
C12—C16	1.428 (3)	C21—H21	0.9300
C12—C13	1.428 (2)	O1—H1	0.8797
C12—Fe1	2.0234 (17)		
			
O1—C1—C2	115.0 (2)	C18—C17—H17	126.2
O1—C1—H1A	108.5	Fe1—C17—H17	125.6
C2—C1—H1A	108.5	C19—C18—C17	107.0 (2)
O1—C1—H1B	108.5	C19—C18—Fe1	70.27 (13)
C2—C1—H1B	108.5	C17—C18—Fe1	69.23 (12)
H1A—C1—H1B	107.5	C19—C18—H18	126.5
O2—C2—C1	106.55 (19)	C17—C18—H18	126.5
O2—C2—H2A	110.4	Fe1—C18—H18	125.6
C1—C2—H2A	110.4	C20—C19—C18	109.0 (2)
O2—C2—H2B	110.4	C20—C19—Fe1	70.13 (12)
C1—C2—H2B	110.4	C18—C19—Fe1	69.93 (13)
H2A—C2—H2B	108.6	C20—C19—H19	125.5
O2—C3—C4	123.84 (18)	C18—C19—H19	125.5
O2—C3—C8	115.86 (15)	Fe1—C19—H19	126.0
C4—C3—C8	120.29 (19)	C21—C20—C19	108.6 (2)
C5—C4—C3	120.1 (2)	C21—C20—Fe1	69.88 (13)
C5—C4—H4	119.9	C19—C20—Fe1	70.12 (12)
C3—C4—H4	119.9	C21—C20—H20	125.7
C6—C5—C4	120.83 (19)	C19—C20—H20	125.7
C6—C5—H5	119.6	Fe1—C20—H20	125.9
C4—C5—H5	119.6	C20—C21—C17	107.8 (2)
C5—C6—C7	119.1 (2)	C20—C21—Fe1	70.42 (13)
C5—C6—H6	120.4	C17—C21—Fe1	69.49 (13)
C7—C6—H6	120.4	C20—C21—H21	126.1
C6—C7—C8	122.2 (2)	C17—C21—H21	126.1
C6—C7—H7	118.9	Fe1—C21—H21	125.6
C8—C7—H7	118.9	C1—O1—H1	109.00
C7—C8—C3	117.44 (17)	C3—O2—C2	119.45 (16)
C7—C8—C9	117.55 (16)	C12—Fe1—C17	123.79 (10)
C3—C8—C9	124.98 (16)	C12—Fe1—C16	41.28 (8)
C10—C9—C8	130.69 (16)	C17—Fe1—C16	160.08 (11)
C10—C9—H9	114.7	C12—Fe1—C21	160.77 (10)
C8—C9—H9	114.7	C17—Fe1—C21	40.55 (11)
C9—C10—C11	120.68 (15)	C16—Fe1—C21	157.08 (11)
C9—C10—H10	119.7	C12—Fe1—C18	107.13 (8)
C11—C10—H10	119.7	C17—Fe1—C18	40.89 (11)
O3—C11—C12	119.67 (16)	C16—Fe1—C18	122.76 (9)
O3—C11—C10	122.17 (15)	C21—Fe1—C18	68.19 (10)
C12—C11—C10	118.15 (15)	C12—Fe1—C20	157.48 (9)
C16—C12—C13	107.29 (15)	C17—Fe1—C20	67.31 (11)
C16—C12—C11	128.45 (16)	C16—Fe1—C20	121.80 (10)
C13—C12—C11	123.87 (16)	C21—Fe1—C20	39.70 (11)
C16—C12—Fe1	69.50 (10)	C18—Fe1—C20	67.32 (10)
C13—C12—Fe1	70.17 (10)	C12—Fe1—C19	122.09 (9)
C11—C12—Fe1	120.02 (11)	C17—Fe1—C19	67.37 (11)
C14—C13—C12	108.35 (18)	C16—Fe1—C19	107.33 (10)
C14—C13—Fe1	70.47 (12)	C21—Fe1—C19	67.07 (10)
C12—C13—Fe1	68.71 (10)	C18—Fe1—C19	39.81 (10)
C14—C13—H13	125.8	C20—Fe1—C19	39.75 (10)
C12—C13—H13	125.8	C12—Fe1—C13	41.12 (7)
Fe1—C13—H13	126.6	C17—Fe1—C13	108.66 (9)
C15—C14—C13	107.80 (17)	C16—Fe1—C13	68.82 (8)
C15—C14—Fe1	69.64 (12)	C21—Fe1—C13	124.62 (9)
C13—C14—Fe1	69.33 (11)	C18—Fe1—C13	123.29 (9)
C15—C14—H14	126.1	C20—Fe1—C13	160.23 (9)
C13—C14—H14	126.1	C19—Fe1—C13	158.58 (9)
Fe1—C14—H14	126.5	C12—Fe1—C15	68.40 (8)
C14—C15—C16	109.06 (18)	C17—Fe1—C15	158.41 (11)
C14—C15—Fe1	70.27 (11)	C16—Fe1—C15	40.51 (7)
C16—C15—Fe1	68.89 (10)	C21—Fe1—C15	122.65 (10)
C14—C15—H15	125.5	C18—Fe1—C15	159.19 (11)
C16—C15—H15	125.5	C20—Fe1—C15	108.56 (10)
Fe1—C15—H15	127.0	C19—Fe1—C15	124.01 (11)
C15—C16—C12	107.48 (18)	C13—Fe1—C15	67.59 (9)
C15—C16—Fe1	70.60 (11)	C12—Fe1—C14	68.62 (7)
C12—C16—Fe1	69.22 (9)	C17—Fe1—C14	123.28 (11)
C15—C16—H16	126.3	C16—Fe1—C14	68.40 (9)
C12—C16—H16	126.3	C21—Fe1—C14	108.71 (9)
Fe1—C16—H16	125.5	C18—Fe1—C14	159.13 (11)
C21—C17—C18	107.6 (2)	C20—Fe1—C14	124.38 (9)
C21—C17—Fe1	69.96 (13)	C19—Fe1—C14	159.79 (10)
C18—C17—Fe1	69.88 (12)	C13—Fe1—C14	40.20 (8)
C21—C17—H17	126.2	C15—Fe1—C14	40.09 (9)
			
O1—C1—C2—O2	56.9 (3)	C15—C16—Fe1—C14	−36.56 (14)
O2—C3—C4—C5	−177.42 (19)	C12—C16—Fe1—C14	81.76 (12)
C8—C3—C4—C5	0.9 (3)	C20—C21—Fe1—C12	−159.7 (2)
C3—C4—C5—C6	0.3 (3)	C17—C21—Fe1—C12	−41.1 (3)
C4—C5—C6—C7	−0.9 (4)	C20—C21—Fe1—C17	−118.6 (2)
C5—C6—C7—C8	0.3 (3)	C20—C21—Fe1—C16	43.1 (3)
C6—C7—C8—C3	0.9 (3)	C17—C21—Fe1—C16	161.7 (2)
C6—C7—C8—C9	−177.20 (19)	C20—C21—Fe1—C18	−80.31 (17)
O2—C3—C8—C7	177.02 (16)	C17—C21—Fe1—C18	38.30 (16)
C4—C3—C8—C7	−1.5 (3)	C17—C21—Fe1—C20	118.6 (2)
O2—C3—C8—C9	−5.1 (3)	C20—C21—Fe1—C19	−37.13 (16)
C4—C3—C8—C9	176.43 (17)	C17—C21—Fe1—C19	81.48 (17)
C7—C8—C9—C10	179.68 (19)	C20—C21—Fe1—C13	163.45 (14)
C3—C8—C9—C10	1.8 (3)	C17—C21—Fe1—C13	−77.94 (17)
C8—C9—C10—C11	−178.11 (16)	C20—C21—Fe1—C15	79.63 (17)
C9—C10—C11—O3	6.4 (3)	C17—C21—Fe1—C15	−161.76 (16)
C9—C10—C11—C12	−172.95 (16)	C20—C21—Fe1—C14	121.70 (15)
O3—C11—C12—C16	175.64 (18)	C17—C21—Fe1—C14	−119.69 (16)
C10—C11—C12—C16	−5.0 (3)	C19—C18—Fe1—C12	−119.90 (14)
O3—C11—C12—C13	−12.5 (3)	C17—C18—Fe1—C12	122.21 (15)
C10—C11—C12—C13	166.86 (16)	C19—C18—Fe1—C17	117.9 (2)
O3—C11—C12—Fe1	−97.67 (18)	C19—C18—Fe1—C16	−77.36 (17)
C10—C11—C12—Fe1	81.67 (18)	C17—C18—Fe1—C16	164.76 (15)
C16—C12—C13—C14	0.3 (2)	C19—C18—Fe1—C21	79.90 (17)
C11—C12—C13—C14	−173.01 (16)	C17—C18—Fe1—C21	−37.99 (16)
Fe1—C12—C13—C14	−59.52 (14)	C19—C18—Fe1—C20	36.86 (16)
C16—C12—C13—Fe1	59.86 (12)	C17—C18—Fe1—C20	−81.03 (17)
C11—C12—C13—Fe1	−113.49 (16)	C17—C18—Fe1—C19	−117.9 (2)
C12—C13—C14—C15	−0.8 (2)	C19—C18—Fe1—C13	−162.11 (14)
Fe1—C13—C14—C15	−59.22 (15)	C17—C18—Fe1—C13	80.01 (17)
C12—C13—C14—Fe1	58.43 (13)	C19—C18—Fe1—C15	−45.8 (3)
C13—C14—C15—C16	0.9 (2)	C17—C18—Fe1—C15	−163.6 (2)
Fe1—C14—C15—C16	−58.08 (15)	C19—C18—Fe1—C14	165.3 (2)
C13—C14—C15—Fe1	59.02 (14)	C17—C18—Fe1—C14	47.4 (3)
C14—C15—C16—C12	−0.7 (2)	C21—C20—Fe1—C12	162.6 (2)
Fe1—C15—C16—C12	−59.65 (13)	C19—C20—Fe1—C12	43.0 (3)
C14—C15—C16—Fe1	58.92 (15)	C21—C20—Fe1—C17	38.22 (16)
C13—C12—C16—C15	0.2 (2)	C19—C20—Fe1—C17	−81.40 (18)
C11—C12—C16—C15	173.19 (17)	C21—C20—Fe1—C16	−161.77 (14)
Fe1—C12—C16—C15	60.53 (14)	C19—C20—Fe1—C16	78.61 (18)
C13—C12—C16—Fe1	−60.29 (12)	C19—C20—Fe1—C21	−119.6 (2)
C11—C12—C16—Fe1	112.66 (17)	C21—C20—Fe1—C18	82.71 (17)
C21—C17—C18—C19	−0.4 (3)	C19—C20—Fe1—C18	−36.91 (16)
Fe1—C17—C18—C19	−60.44 (16)	C21—C20—Fe1—C19	119.6 (2)
C21—C17—C18—Fe1	60.05 (15)	C21—C20—Fe1—C13	−43.9 (3)
C17—C18—C19—C20	0.3 (3)	C19—C20—Fe1—C13	−163.5 (2)
Fe1—C18—C19—C20	−59.43 (17)	C21—C20—Fe1—C15	−119.11 (16)
C17—C18—C19—Fe1	59.78 (15)	C19—C20—Fe1—C15	121.27 (16)
C18—C19—C20—C21	−0.2 (3)	C21—C20—Fe1—C14	−77.54 (18)
Fe1—C19—C20—C21	−59.47 (16)	C19—C20—Fe1—C14	162.84 (15)
C18—C19—C20—Fe1	59.30 (16)	C20—C19—Fe1—C12	−162.04 (15)
C19—C20—C21—C17	−0.1 (3)	C18—C19—Fe1—C12	77.91 (17)
Fe1—C20—C21—C17	−59.70 (16)	C20—C19—Fe1—C17	81.24 (18)
C19—C20—C21—Fe1	59.62 (16)	C18—C19—Fe1—C17	−38.82 (16)
C18—C17—C21—C20	0.3 (3)	C20—C19—Fe1—C16	−119.22 (16)
Fe1—C17—C21—C20	60.29 (16)	C18—C19—Fe1—C16	120.73 (15)
C18—C17—C21—Fe1	−60.00 (15)	C20—C19—Fe1—C21	37.08 (17)
C4—C3—O2—C2	−14.0 (3)	C18—C19—Fe1—C21	−82.97 (17)
C8—C3—O2—C2	167.58 (17)	C20—C19—Fe1—C18	120.1 (2)
C1—C2—O2—C3	−179.56 (19)	C18—C19—Fe1—C20	−120.1 (2)
C16—C12—Fe1—C17	162.26 (14)	C20—C19—Fe1—C13	164.7 (2)
C13—C12—Fe1—C17	−79.57 (15)	C18—C19—Fe1—C13	44.7 (3)
C11—C12—Fe1—C17	38.85 (19)	C20—C19—Fe1—C15	−77.83 (18)
C13—C12—Fe1—C16	118.17 (15)	C18—C19—Fe1—C15	162.12 (14)
C11—C12—Fe1—C16	−123.41 (19)	C20—C19—Fe1—C14	−44.8 (3)
C16—C12—Fe1—C21	−166.8 (3)	C18—C19—Fe1—C14	−164.9 (2)
C13—C12—Fe1—C21	−48.6 (3)	C14—C13—Fe1—C12	119.78 (17)
C11—C12—Fe1—C21	69.8 (3)	C14—C13—Fe1—C17	−119.84 (15)
C16—C12—Fe1—C18	120.48 (13)	C12—C13—Fe1—C17	120.38 (14)
C13—C12—Fe1—C18	−121.35 (13)	C14—C13—Fe1—C16	81.19 (13)
C11—C12—Fe1—C18	−2.93 (17)	C12—C13—Fe1—C16	−38.59 (10)
C16—C12—Fe1—C20	48.6 (3)	C14—C13—Fe1—C21	−77.69 (16)
C13—C12—Fe1—C20	166.7 (2)	C12—C13—Fe1—C21	162.52 (13)
C11—C12—Fe1—C20	−74.9 (3)	C14—C13—Fe1—C18	−162.72 (14)
C16—C12—Fe1—C19	79.55 (14)	C12—C13—Fe1—C18	77.50 (14)
C13—C12—Fe1—C19	−162.28 (13)	C14—C13—Fe1—C20	−45.2 (3)
C11—C12—Fe1—C19	−43.86 (18)	C12—C13—Fe1—C20	−164.9 (3)
C16—C12—Fe1—C13	−118.17 (15)	C14—C13—Fe1—C19	164.7 (2)
C11—C12—Fe1—C13	118.42 (19)	C12—C13—Fe1—C19	44.9 (3)
C16—C12—Fe1—C15	−37.96 (12)	C14—C13—Fe1—C15	37.43 (12)
C13—C12—Fe1—C15	80.21 (12)	C12—C13—Fe1—C15	−82.36 (12)
C11—C12—Fe1—C15	−161.37 (17)	C12—C13—Fe1—C14	−119.78 (17)
C16—C12—Fe1—C14	−81.18 (13)	C14—C15—Fe1—C12	−82.03 (13)
C13—C12—Fe1—C14	36.99 (12)	C16—C15—Fe1—C12	38.66 (13)
C11—C12—Fe1—C14	155.40 (16)	C14—C15—Fe1—C17	46.7 (3)
C21—C17—Fe1—C12	164.90 (14)	C16—C15—Fe1—C17	167.3 (3)
C18—C17—Fe1—C12	−76.62 (16)	C14—C15—Fe1—C16	−120.69 (19)
C21—C17—Fe1—C16	−158.9 (3)	C14—C15—Fe1—C21	80.23 (16)
C18—C17—Fe1—C16	−40.5 (4)	C16—C15—Fe1—C21	−159.08 (15)
C18—C17—Fe1—C21	118.5 (2)	C14—C15—Fe1—C18	−163.4 (2)
C21—C17—Fe1—C18	−118.5 (2)	C16—C15—Fe1—C18	−42.7 (3)
C21—C17—Fe1—C20	−37.43 (15)	C14—C15—Fe1—C20	121.75 (14)
C18—C17—Fe1—C20	81.05 (16)	C16—C15—Fe1—C20	−117.56 (15)
C21—C17—Fe1—C19	−80.67 (17)	C14—C15—Fe1—C19	163.00 (13)
C18—C17—Fe1—C19	37.81 (14)	C16—C15—Fe1—C19	−76.31 (17)
C21—C17—Fe1—C13	121.85 (15)	C14—C15—Fe1—C13	−37.53 (12)
C18—C17—Fe1—C13	−119.67 (14)	C16—C15—Fe1—C13	83.16 (14)
C21—C17—Fe1—C15	45.7 (3)	C16—C15—Fe1—C14	120.69 (19)
C18—C17—Fe1—C15	164.2 (2)	C15—C14—Fe1—C12	81.45 (12)
C21—C17—Fe1—C14	79.81 (18)	C13—C14—Fe1—C12	−37.80 (11)
C18—C17—Fe1—C14	−161.71 (14)	C15—C14—Fe1—C17	−161.33 (14)
C15—C16—Fe1—C12	−118.31 (19)	C13—C14—Fe1—C17	79.42 (16)
C15—C16—Fe1—C17	−166.3 (3)	C15—C14—Fe1—C16	36.93 (12)
C12—C16—Fe1—C17	−48.0 (3)	C13—C14—Fe1—C16	−82.33 (12)
C15—C16—Fe1—C21	50.5 (3)	C15—C14—Fe1—C21	−118.83 (14)
C12—C16—Fe1—C21	168.9 (2)	C13—C14—Fe1—C21	121.91 (14)
C15—C16—Fe1—C18	163.34 (15)	C15—C14—Fe1—C18	163.5 (2)
C12—C16—Fe1—C18	−78.34 (15)	C13—C14—Fe1—C18	44.2 (3)
C15—C16—Fe1—C20	81.43 (17)	C15—C14—Fe1—C20	−77.64 (16)
C12—C16—Fe1—C20	−160.26 (12)	C13—C14—Fe1—C20	163.10 (13)
C15—C16—Fe1—C19	122.47 (15)	C15—C14—Fe1—C19	−44.6 (3)
C12—C16—Fe1—C19	−119.21 (12)	C13—C14—Fe1—C19	−163.8 (2)
C15—C16—Fe1—C13	−79.87 (15)	C15—C14—Fe1—C13	119.25 (17)
C12—C16—Fe1—C13	38.44 (10)	C13—C14—Fe1—C15	−119.25 (17)
C12—C16—Fe1—C15	118.31 (19)		

### Hydrogen-bond geometry (Å, º)

**Table:**

*D*—H···*A*	*D*—H	H···*A*	*D*···*A*	*D*—H···*A*
O1—H1···O3^i^	0.88	1.98	2.841 (2)	165
C7—H7···O3^ii^	0.93	2.56	3.409 (3)	152

Symmetry codes: (i) −*x*+1, −*y*, −*z*+1; (ii) −*x*+1, −*y*, −*z*+2.
